# Evaluation of *ex vivo* melanogenic response to UVB, UVA, and visible light in facial melasma and unaffected adjacent skin^⋆^^⋆⋆^

**DOI:** 10.1016/j.abd.2020.02.015

**Published:** 2020-09-17

**Authors:** Giovana Piteri Alcantara, Ana Cláudia Cavalcante Esposito, Thainá Oliveira Felicio Olivatti, Melissa Mari Yoshida, Hélio Amante Miot

**Affiliations:** a Department of Dermatology and Radiotherapy, Faculty of Medicine, Universidade Estadual Paulista, Botucatu, SP, Brazil; b Graduate Program in Pathology, Faculty of Medicine, Universidade Estadual Paulista, Botucatu, SP, Brazil; c Faculty of Medicine, Universidade Estadual Paulista, Botucatu, SP, Brazil

**Keywords:** Melanosis, Photobiology, Ultraviolet rays

## Abstract

**Background:**

The independent role of solar radiation in the differential melanogenesis between melasma and adjacent skin is unknown.

**Objectives:**

To assess the melanogenic responses of skin with facial melasma and of the adjacent skin to UVB, UVA, and visible light, in an *ex vivo* model.

**Methods:**

This was a quasi-experimental study involving 22 patients with melasma. Facial melasma and adjacent skin samples were collected and stored in DMEM medium, at room temperature. One fragment was placed under the protection from light, while another was exposed to UVB, UVA, and visible light (blue-violet component): 166 mJ/cm^2^, 1.524 J/cm^2^, and 40 J/cm^2^, respectively. Subsequently, all samples were kept for 72 hours in a dark environment and stained by Fontana-Masson to assess basal layer pigmentation, dendrites, and melanin granulation.

**Results:**

Effective melanogenesis was observed in the basal layer in melasma and in the normal adjacent skin after all irradiations (*p* < 0.01), with the following median increment: UVB (4.7% *vs*. 8.5%), UVA (9.5% *vs*. 9.9%), and visible light (6.8% *vs*. 11.7%), with no significant difference between anatomical sites. An increase in melanin granulation (coarser melanosomes) was observed only after irradiation with UVA and only in the skin with melasma (*p* = 0.05). An increase in the melanocyte dendrite count induced by UVB radiation was observed in both anatomical sites (*p* ≤ 0.05).

**Study limitations:**

Use of an *ex vivo* model, with independent irradiation regimes for UVB, UVA, and visible light.

**Conclusions:**

Melanogenesis induced by UVB, UVA, and visible light was observed both in melasma and in the adjacent skin. The morphological patterns suggest that different irradiations promote individualized responses on the skin with melasma.

## Introduction

Melasma is a chronic and acquired hypermelanosis resulting from a dysfunction of melanogenesis: increased production, transfer, and greater maturity of melanosomes.^1^ It is a frequent condition in the dermatological routine; according to a study by the Brazilian Society of Dermatology published in 2018, it accounts for 3.6% of dermatological consultations.^2^ It affects mainly women of childbearing age (25–50 years) and intermediate phototypes (III–V). The clinical lesions of melasma are macules, commonly symmetrical, hyperchromic, well-defined, and located in anatomical sites exposed to the sun, especially the face.^3^ Its pathophysiology is still not completely understood, but there is an interaction between genetics (autosomal dominant inheritance), hormonal factors, and exposure to solar radiation, leading to greater focal melanogenesis.^3^, ^4^, ^5^, ^6^, ^7^

Phenotypic changes in the skin with melasma result from both morphological (enlarged melanocytes and prominent dendrites) and dysfunctional epidermal alterations, resulting from the complex interaction between keratinocytes, melanocytes, and components of the upper dermis.^8^

There is a clear relationship between sun exposure and melasma worsening, due to its exclusive occurrence in photo exposed areas, prevalence in professionals who are exposed to the sun, and better therapeutic response associated with photoprotection.^9^, ^10^ Even with evidence of the role of solar radiation in the clinical history of the disease, the individualized response pattern to the different bands of solar radiation (UVB, UVA, and visible light [VL]) of melasma in comparison with normal adjacent skin is not known.

Knowledge of the different influences of solar radiation in the melanogenesis of melasma can lead to a better understanding of the pathophysiology of the disease, as well as to the development of new treatment options and optimized photo protection strategies.

This study aimed to determine the melanogenic response of facial melasma in comparison with the normal adjacent skin after irradiation with UVA, UVB, or VL in an *ex vivo* model.

## Material and methods

This was a *quasi*-experimental *ex vivo* study of skin fragments of 22 patients with facial melasma, diagnosed by an experienced dermatologist. Participants were selected among patients attended at the dermatology service of Hospital das Clínicas, Faculty of Medicine de Botucatu, SP, Brazil, between September/2018 and January/2019. The project was approved by the research ethics committee (No. 2,700,889) and all participants signed an informed consent form before inclusion.

Adult patients (>18 years old), of both sexes, with phototypes III–V, with facial melasma and without treatment, except for sunscreen for at least 30 days, were eligible for the study. The phototype restriction was based on the fact that phototypes I and II represent less than 10% of cases of melasma and do not show satisfactory melanogenesis after experimental irradiation.^3^, ^11^ There is no description of melasma in phototype VI.

Patients with other facial dermatoses, photosensitive dermatoses, collagenoses, blood dyscrasias, users of anticoagulant medication, those who were immunosuppressed, and pregnant or lactating women were not included in the study. Sampling was carried out by convenience, recruiting consecutive consenting patients during medical consultations at the institution.

Sample material collection occurred in a standardized way and under a sterile procedure. Two samples from each participant were collected: skin with melasma and normal adjacent skin, up to 2 cm apart, under local infiltrative anesthesia with 2% lidocaine with vasoconstrictor. The removal included the reticular dermis, with a 3 mm punch and 6–0 nylon monofilament suture. Peripheral areas of the face were preferred, in order to minimize unsightly scars.

Samples were sectioned longitudinally in two parts and stored in 10 mL of DMEM (Dulbecco’s Modified Eagle’s Medium, high glucose – D5796, Sigma Inc., United Kingdom) in a sterile transparent plastic bottle and kept at room temperature, according to the protocol set forth by Olivatti.^12^ A part of the fragment was placed immediately in the dark, while the other was exposed to radiation, for a total UVB, UVA, and VL dose (effective in the tissue) of 166 mJ/cm^2^, 1.524 J/cm^2^, and 40 J/cm^2^ (blue-violet light component), respectively. The fragments were irradiated with artificial sources of UVB (230 µW/cm^2^; source: FS72T12/UVB/HO) for 12 min, UVA (1270 µW/cm^2^; source: Phillips TL 100W/10R) for 6 min, and LED light (110 mW/cm^2^ in the blue-violet band; source: GBRLUX 200W) for 20 min, at a standardized distance of 10 cm. The absorbance of the plastic vials was around 50% for all radiations. The allocation of participants in each type of irradiation was consecutive (not randomized).

After irradiation, both fragments (irradiated and non-irradiated) were cultured in a dark setting. After 72 hours, the samples were removed from the culture medium and preserved in 10% buffered formalin for 36 hours. Subsequently, they were dehydrated in 70% alcohol for at least 12 hours before embedding in paraffin and Fontana-Masson (FM) staining.

To estimate the melanization of the fragments, four photographs of central and interfolicular areas of the samples stained by FM were captured, favoring hotspot areas; the images were saved in. JPG format (1264 × 681 pixels). During the entire capture and analysis process, the investigators were blinded to the anatomical sites or type and irradiation regime.

The selected images were analyzed using ImageJ 1.43, from the manual selection of the basal layer, followed by thresholding using the “red” channel.^13^

The main outcome of the study was the percentage of melanization of the basal layer, compared between groups (melasma *vs.* adjacent normal skin) for each radiation (UVB, UVA, or VL). The percentage of melanin in the superficial dermis, dendrite count, and melanosome granulation were also evaluated (score: 1+, fine granulation; 2+, moderate granulation; 3+, coarse granulation) in the sampled areas.

The mean value of the percentage of melanin in the basal layer and in the upper dermis, dendrite count, and granulation scores were tabulated for each specimen. The comparison groups were assessed by a generalized linear mixed effects model, adjusted for phototype (robust analysis) with an unstructured covariance matrix and Šidák’s *post-hoc* correction.^14^ The probability distributions varied for the type of variable analyzed (ordinal and gamma).

The sample was sized after a pretest with radiation dosimetry that led to the expectation of a 10% increase in the percentage of melanization in the basal layer evaluated by digital image analysis of the slides stained by FM, considering alpha of 5% and power of 20%.^15^

The data were tabulated in MSExcel 2013 and analyzed in IBM SPSS v. 25. Two-tailed *p*-values ≤0.05 were considered significant.

## Results

A total of 89 skin fragments from 22 patients with facial melasma were evaluated (one participant volunteered four samples in order to test all irradiations). The mean age (standard deviation) of the participants was 42.1 (10.4) years, 21 of whom were female, with the following phototypes: III (46%), IV (27%), and V (27%). Three culture fragments were contaminated (UVA, normal adjacent skin, pre-irradiation; UVB, melasmic skin, post-irradiation; and UVB, normal adjacent skin, post-irradiation) and were not included in the analysis of the results.

Patients whose samples were submitted to UVB, UVA, and VL irradiation had the following phototypes, respectively: III (33% *vs*. 37% vs. 0%), IV (44% *vs*. 37% *vs*. 71%), and VL (22% *vs*. 25% *vs*. 28%) (*p* = 0.35).

**Figure 1:**
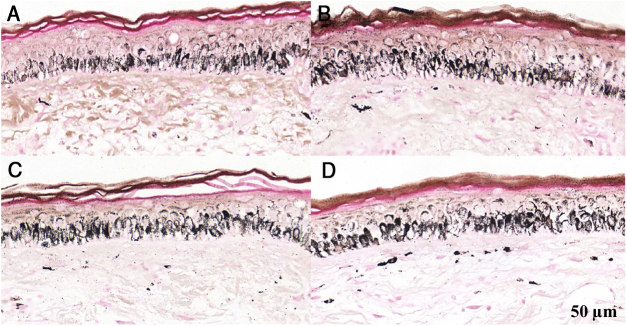
Histological sections of skin with melasma and normal adjacent skin samples stained with Fontana-Masson before and after irradiation with 166 mJ/cm^2^ UVB, revealing an increase in the basal melanin density in the different samples. (A) Normal adjacent skin before UVB irradiation; (B) normal adjacent skin after UVB irradiation; (C) skin with melasma before UVB irradiation; (D) skin with melasma after UVB irradiation.

**Figure 2:**
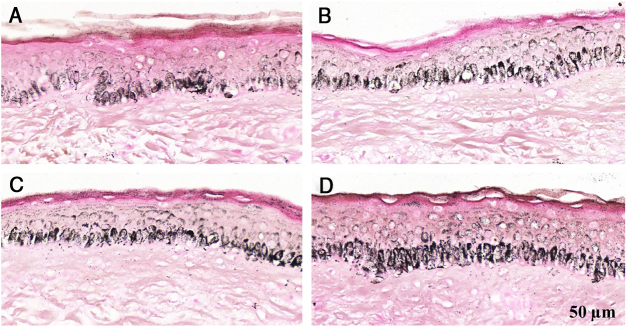
Histological sections of skin with melasma and normal adjacent skin samples stained with Fontana-Masson before and after irradiation with 1.524 mJ/cm^2^ UVB, revealing an increase in the basal melanin density in the different samples. (A), normal adjacent skin before UVB irradiation; (B), normal adjacent skin after UVB irradiation; (C), skin with melasma before UVB irradiation; (D), skin with melasma after UVB irradiation.

**Figure 3:**
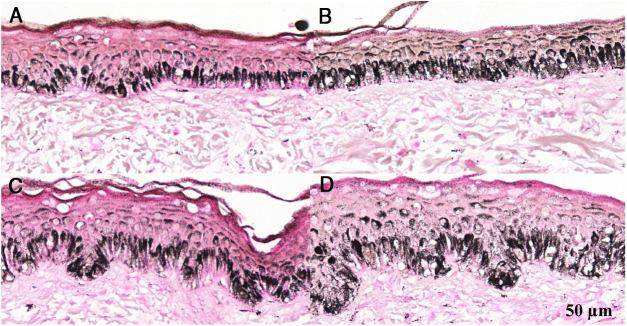
Histological sections of skin with melasma and normal adjacent skin samples stained with Fontana-Masson before and after irradiation with 40 mJ/cm^2^ visible light (VL), revealing an increase in the basal melanin density between the samples. (A), normal adjacent skin before VL irradiation; (B), normal adjacent skin after VL irradiation; (C), skin with melasma before VL irradiation; (D), skin with melasma after VL irradiation.

Melanogenesis was observed in melasma and in normal adjacent skin (*p* < 0.01) after all irradiations (Figure 1, Figure 2, Figure 3, Table 1): median UVB increment (4.7% *vs*. 8.5%), UVA (9.5% *vs*. 9.9%), and VL (6.8% *vs*. 11.7%), with no significant difference in the comparison between anatomical sites.

**Table 1:** Median and quartiles (p25–p75) of the percentage of melanin in the basal layer and in the upper dermis, dendrite count, and melanin granulation in the histological sections of skin with melasma and normal adjacent skin, before and after (pre and post) irradiation with UVB, UVA, and visible light (VL). Data analysis adjusted by phototype.

Irradiation	Variable	Facial melasma			Normal adjacent skin			*p* (time × group)**
		Pre	Post	p*	Pre	Post	p*	
UVB	MelBasal	64 (60–65)	65 (65–68)	<0.01	54 (53–56)	56 (55–62)	<0.01	0.09
	MelUD	44 (30–77)	45 (38–97)	0.24	44 (32–71)	67 (41–87)	0.04	0.84
	Dendrites	6 (2–8)	10 (8–18)	<0.01	6 (3–14)	11 (7–16)	0.05	0.59
	GranMelanos	2.0 (1.5–2.1)	1.6 (1.3–2.0)	0.11	1.9 (1.4–2.0)	1.9 (1.6–2.0)	0.80	0.98
UVA	MelBasal	62 (54–66)	70 (57–72)	<0.01	56 (49–61)	57 (50–63)	<0.01	0.06
	MelUD	41 (18–88)	86 (36–144)	0.05	45 (27–90)	74 (34–204)	0.50	0.88
	Dendrites	8 (4–11)	8 (2–22)	0.92	3 (2–5)	5 (3–9)	0.26	0.53
	GranMelanos	1.0 (1.0–1.8)	2.0 (1.8–2.1)	0.05	1.3 (1.3–2.0)	1.3 (1.1–1.9)	0.34	0.03
Visible light	MelBasal	73 (59–74)	75 (69–80)	<0.01	64 (56–66)	70 (61–75)	<0.01	0.09
	MelUD	62 (54–72)	52 (45–83)	0.11	66 (59–83)	75 (53–127)	0.34	0.21
	Dendrites	2 (1–4)	4 (2–14)	0.11	1 (0–3)	4 (1–5)	0.06	0.38
	GranMelanos	1.4 (1.0–2.0)	1.5 (1.0–2.3)	0.11	1.5 (1.0–2.0)	1.5 (1.0–2.0)	0.80	0.98

MelBasal, percentage of melanin in the basal layer of the epidermis; MelUD, percentage of melanin in the upper dermis (×100); GranMelanos: granulation score of melanosomes of the epidermis (1+, fine granulation; 2+, moderate granulation; 3+, coarse granulation), mean of four fields.

* *p*-value, pre *vs*. post (adjusted by phototype).

** *p*-value, melasma *vs*. normal adjacent skin depending on the response to irradiation (adjusted by phototype).

When adjusted for phototype, the dendrite count increased (68.8%) only after irradiation with UVB in both anatomical sites (Table 1). An increase in melanin granulation (coarser melanosomes) was observed only after irradiation with UVA and only in the skin with melasma (*p* = 0.05). An increase was observed in the percentage of melanin in the upper dermis in the adjacent skin after irradiation with UVB, and in melasmic skin after irradiation with UVA (*p* ≤ 0.05).

## Discussion

Effective epidermal melanogenesis was observed in skin models (*ex vivo*) submitted to UVB, UVA, and VL, with no quantitative difference between the anatomical sites (facial melasma and normal adjacent skin), supporting the importance of broad spectrum photoprotection in the treatment of pigmentary dermatoses, such as melasma.^16^

The radiation doses tested, although independent, were equivalent to approximately 6–12 min of sun exposure at midday, in the summer, in countryside Brazil (latitude: 22º53′09″S; longitude: 48º26′42″W; altitude: 804 m), on a cloudless day, as measured on December 9, 2018, by the researchers. However, the levels of solar UVA and VL reach an energy plateau from 9 am to 6 pm, while the UVB peaks between 11 am and 3 pm, both in summer and in winter. This may justify the report of melasma worsening after brief unprotected exposures outside peak UVB hours, or even after long exposures with a photoprotector (*e.g.*, during a traffic jam), since sunscreens do not completely block solar radiation.^17^ UVB, UVA, and VL radiation measured in closed environments (*e.g*., from cell phone and computer screens, or fluorescent lamps) are negligible for the purposes of melanogenesis (data not shown).

VL has a wide spectrum of wavelengths (400–700 nm) that do not act homogeneously on pigmentation. A study carried out in France tested the spectrum of blue-violet light (400–500 nm) in comparison with the spectrum of red light (620–750 nm); those authors observed no pigmentation induced by the latter, justifying the choice of the blue-violet band for the present study.^18^

Only opaque sunscreens have effective protection against VL; the majority of them also perform better in UVA protection. In a Brazilian survey with 41 opaque sunscreens, 63% blocked >99.9% of UVA, and 63% blocked >99.9% of blue-violet light transmittance. While the percentage was the same, it was not overlapped: 31% of opaque filters that blocked >99.9% of the VL did not present the same performance for UVA.^17^ The present study is the first to highlight the role of VL in the melanogenesis of patients with facial melasma.

No differential melanogenesis was observed between melasma and adjacent normal skin at the doses tested, which does not exclude the possibility that melasmic skin is sensitive to lower doses of irradiation or responds differently to repeated exposures. The greater basal pigmentation of melasma itself can also present some resistance to melanogenesis induced by chronic exposure to UVR and VL. Furthermore, there is greater epidermal retention of melanin in melasma, and its melanogenesis depends on several other dermal changes, which favor the maintenance of pigmentation to the detriment of melanogenic induction by UVR and VL.^4^

Melanogenesis occurs unevenly, depending on the type and dose of radiation. UVB causes erythema that persists for days to weeks (depending on the phototype) and pigmentation that evolves to tanning. UVA causes erythema in four hours and immediate pigmentation that evolves with lasting tanning. In VL, erythema is less prominent and may occur early (within two hours). Pigmentation is sustained with progression to tanning, but only in higher phototypes.^19^, ^20^, ^21^

Every melanogenic response occurs in a dose-dependent manner. However, it is essential to consider the phototypes when analyzing the performance of different radiations, since melanogenesis is different in the higher phototypes (III–V), causing greater pigmentation with evolution to tanning in a dose-dependent manner. In turn, in lower phototypes (I–I) irradiation promotes less stimulation. In an experimental study, UVA and VL did not induce melanogenesis in patients with phototype II.^11^, ^22^

Human melanogenesis is a complex process in which different interactions occur in an organized manner to protect skin structures and to regulate cycles that are essential for survival, such as the circadian cycle. Melanocytes are a network with their own sense of ambient light, responding according to the aforementioned objectives.^23^

Different characteristics were observed in the melanogenic response when comparing anatomical sites and radiations. The formation of dendrites occurs in the G2 phase of melanocyte growth and is an important sign of melanogenic response; in the present study, it was mainly induced by the irradiation with the greatest mutagenic potential (UVB). Although melanogenesis is perceived in a similar way in both anatomical sites, the formation of dendrites can indicate a greater persistence of the phenomenon and greater efficiency in the transfer of melanosomes in the epidermis.^11^

Melasmic skin is also characterized by the presence of more mature melanosomes, equivalent to what occurs in more pigmented populations. In the present experiment, UVA irradiation promoted more evident granulation than the other tested radiations exclusively on skin with melasma, showing that, in melasma, melanogenesis is more intense and resembles the most pigmented phenotypes. In fact, African ancestry was associated with an increased risk of melasma in the Brazilian population, and ancestral genes related to melanogenesis may justify the differentiated pigment response between anatomical sites.^7^

The role of dermal melanin remains unclear in melasma; one of the hypotheses is that the architectural dysfunction of the basement membrane and the autophagy of the melanocytes of the dermis may cause the presence of dermal melanophages, similar to what occurs in photoaging.^24^, ^25^ In the present study, UVA and UVB promoted a higher rate of melanin in the upper dermis; however, this density was approximately 100 times smaller than that of the epidermis in both anatomical sites, which likely little contributes to the clinical phenotype, but may signal underlying alterations in the upper dermis, basement membrane, and basal layer.

Finally, despite the fundamental role of solar radiation in worsening melasma, no differential expressions of p53 between the melasma and the normal adjacent skin were observed, suggesting that the maintenance of melanogenesis may be due to underlying changes in the upper dermis and epidermis, which induce melanocytic hyperfunction.^4^ Furthermore, the exclusive use of sunscreen, despite its preventive effect, is not sufficient for the complete remission of the condition, which indicates the need for a continuous pathophysiological study in search of treatments that lead to the reversal of the entire process.^9^, ^26^

The present study promoted a preliminary analysis of the independent effects of UVB, UVA, and VL radiation. Further investigations should be conducted in order to elucidate the effect of combinations and dosages of irradiations that mirror environmental conditions. The interaction between UVA and VL is still unknown; they are believed to interact in the same melanin precursor during melanogenesis. This association may result in a more evident behavior of melanogenesis in natural situations, where VL and UVA are expressed together.^20^

This study has limitations inherent to the technique in an *ex vivo* model, which may differ depending on collection and storage. However, this limitation did not prevent the identification of a melanogenic response. Another limitation can be pointed out by the fact that irradiations in *ex vivo* skin models occur with independent wavelengths (UVB, UVA and VL), which is not the case in environmental exposure. In addition to the factors presented above, *ex vivo* models are dissociated from the organism, limiting the interaction between the hormonal, neural, and vasomotor systems.

New experiments should consider low-intensity repeated irradiation models, as well as associated irradiation, to better reproduce the daily exposure of patients. In addition, the inclusion of substances such as tranexamic acid and antioxidants in the prevention of melanogenesis in melasma can be tested.

## Conclusions

An epidermal melanogenic response induced by UVB, UVA, or VL was observed both in skin with melasma and in normal adjacent skin. The morphological patterns related to the photobiology of melanogenesis suggest that different radiations promote individualized responses in the skin with melasma.

## Financial support

10.13039/501100003593CNPq (PIBIC No. 148501/2018-4); FUNADERM (001/2019).

## Authors’ contributions

Giovana Piteri Alcantara: Approval of the final version of the manuscript; conception and planning of the study; elaboration and writing of the manuscript; obtaining, analyzing, and interpreting the data; critical review of the literature; critical review of the manuscript.

Ana Cláudia Cavalcante Esposito: Approval of the final version of the manuscript; conception and planning of the study, elaboration and writing of the manuscript; effective participation in research orientation; critical review of the literature; critical review of the manuscript.

Thainá Oliveira Felicio Olivatti: Approval of the final version of the manuscript; obtaining, analyzing, and interpreting the data; critical review of the manuscript.

Melissa Mari Yoshida: Elaboration and writing of the manuscript; obtaining, analyzing, and interpreting the data; critical review of the manuscript.

Hélio Amante Miot: Statistical analysis; approval of the final version of the manuscript; conception and planning of the study; elaboration and writing of the manuscript; obtaining, analyzing, and interpreting the data; effective participation in research orientation; intellectual participation in propaedeutic and/or therapeutic conduct of studied cases; critical review of the literature; critical review of the manuscript.

## Conflicts of interest

None declared.
